# DEP2: an upgraded comprehensive analysis toolkit for quantitative proteomics data

**DOI:** 10.1093/bioinformatics/btad526

**Published:** 2023-08-25

**Authors:** Zhenhuan Feng, Peiyang Fang, Hui Zheng, Xiaofei Zhang

**Affiliations:** CAS Key Laboratory of Regenerative Biology, Guangdong Provincial Key Laboratory of Stem Cell and Regenerative Medicine, GIBH-HKU Guangdong-Hong Kong Stem Cell and Regenerative Medicine Research Centre, Hong Kong Institute of Science & Innovation, Guangzhou Institutes of Biomedicine and Health, Chinese Academy of Sciences, Guangzhou, Guangdong 510530, China; University of Chinese Academy of Sciences, Beijing 100049, China; Sanquan College, Xinxiang Medical University, Xinxiang, Henan 453003, China; CAS Key Laboratory of Regenerative Biology, Guangdong Provincial Key Laboratory of Stem Cell and Regenerative Medicine, GIBH-HKU Guangdong-Hong Kong Stem Cell and Regenerative Medicine Research Centre, Hong Kong Institute of Science & Innovation, Guangzhou Institutes of Biomedicine and Health, Chinese Academy of Sciences, Guangzhou, Guangdong 510530, China; University of Chinese Academy of Sciences, Beijing 100049, China; Key Laboratory of Biological Targeting Diagnosis, Therapy and Rehabilitation of Guangdong Higher Education Institutes, The Fifth Affiliated Hospital of Guangzhou Medical University, Guangzhou, 510530, China; CAS Key Laboratory of Regenerative Biology, Guangdong Provincial Key Laboratory of Stem Cell and Regenerative Medicine, GIBH-HKU Guangdong-Hong Kong Stem Cell and Regenerative Medicine Research Centre, Hong Kong Institute of Science & Innovation, Guangzhou Institutes of Biomedicine and Health, Chinese Academy of Sciences, Guangzhou, Guangdong 510530, China; University of Chinese Academy of Sciences, Beijing 100049, China; Key Laboratory of Biological Targeting Diagnosis, Therapy and Rehabilitation of Guangdong Higher Education Institutes, The Fifth Affiliated Hospital of Guangzhou Medical University, Guangzhou, 510530, China

## Abstract

**Summary:**

Mass spectrometry (MS)-based proteomics has become the most powerful approach to study the proteome of given biological and clinical samples. Advancements in sample preparation and MS detection have extended the application of proteomics but have also brought new demands on data analysis. Appropriate proteomics data analysis workflow mainly requires quality control, hypothesis testing, functional mining, and visualization. Although there are numerous tools for each process, an efficient and universal tandem analysis toolkit to obtain a quick overall view of various proteomics data is still urgently needed. Here, we present DEP2, an updated version of DEP we previously established, for proteomics data analysis. We amended the analysis workflow by incorporating alternative approaches to accommodate diverse proteomics data, introducing peptide-protein summarization and coupling biological function exploration. In summary, DEP2 is a well-rounded toolkit designed for protein- and peptide-level quantitative proteomics data. It features a more flexible differential analysis workflow and includes a user-friendly Shiny application to facilitate data analysis.

**Availability and implementation:**

DEP2 is available at https://github.com/mildpiggy/DEP2, released under the MIT license. For further information and usage details, please refer to the package website at https://mildpiggy.github.io/DEP2/.

## 1 Introduction

Protein is the executor of life activity for all living organisms. It is now widely acknowledged that the protein expression is not linearly related to transcriptional level (Liu *et al.* 2016). In addition, protein-protein interaction and post-translation modifications (PTMs) such as phosphorylation and ubiquitination regulate protein stability, activity, and localization (Santucci *et al.* 2015, Yue and Lopez 2020). With these variables, it is insufficient to infer proteome merely from transcriptome analysis, although RNA sequencing has made significant advancements. Currently, liquid chromatography–mass spectrometry (MS)-based proteomics is the most powerful approach for studying proteome. Data analysis in MS-based quantitative proteomics study involves two major steps. First, upstream software, such as MaxQuant, is used to identify and quantify matched peptides from spectrums and subsequently aggregate peptide-level abundance into protein abundance (Sinitcyn *et al.* 2018). Second, significant candidates are classified through hypothesis testing for downstream analysis. However, the latter step is restrained by the requirement of bioinformatics analysis, which is often lacking in most wet labs.

Previously, we developed Differential Enrichment analysis of Proteomics data (DEP) (Zhang *et al.* 2018), a package provides a complete pipeline for differential expression/enrichment analysis with moderated *t*-test from limma for proteomics data (Ritchie *et al.* 2015). Although DEP has been widely used by hundreds of labs, it still remains a few drawbacks: (i) DEP is designed for analyzing MaxQuant results and has specific requirements for input file format. Result files from data-independent acquisition (DIA) analysis, such as Spectronaut, DIA-NN are not compatible with DEP; (ii) DEP does not support analysis on PTM-related proteomics (e.g. phosphoproteomics, ubiquitylomics), which relies on both the abundance and site information of modified peptides; and (iii) DEP lacks biological interpretation methods, such as functional enrichment and protein–protein interaction network. While software and packages such as Perseus, ProteoMill, and ProVision have combined statistical test with functional analysis (Tyanova *et al.* 2016, Gallant *et al.* 2020, Ryden *et al.* 2021), most of them show limitations in terms of customization and accessibility. For instance, Perseus only provides a limited selection of imputation and statistical test methods, offering left-shifted imputation combined with *t*-test or ANOVA. On the other hand, ProteoMill and ProVision have strict format requirements for input files.

To provide a comprehensive workflow for proteomics data analysis, we have upgraded DEP to DEP2, with improvements in data compatibility, customizability, and functionality. DEP2 offers a wider range of options throughout the entire workflow and constructs a new analysis pipeline that re-aggregates protein-level abundance from peptide quantification, bypassing the summarization results from upstream software. This pipeline reduces the impact of missing values by implementing an earlier peptide-level imputation (Lazar *et al.* 2016) and also enhances quantitative accuracy through the selection of appropriate aggregation strategy (Sticker *et al.* 2020). Furthermore, we have integrated downstream biological analysis tools to facilitate functional interpretation. Finally, we have redesigned an all-in-one Shiny (https://shiny.posit.co) application under modular design for interactive analysis.

## 2 Results

**Figure 1.**
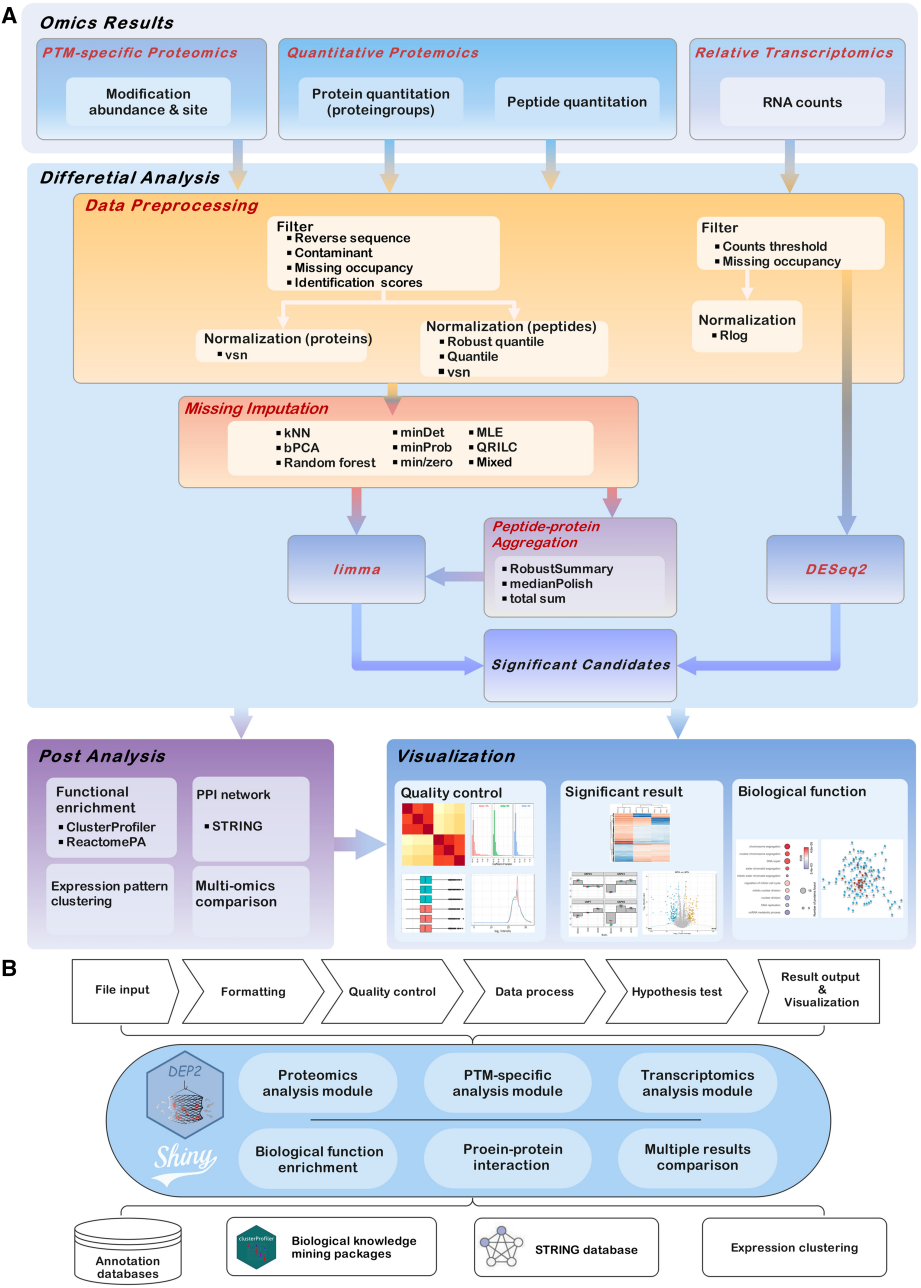
Schematic overviews of DEP2 analyses and built-in application. (A) Functionalities of DEP2 include data processing, statistics test, post-analysis, and result visualization. (B) The Shiny application is modularized, with separate modules for omics pipelines and downstream analyses. The application can be extended through the joint use of modules.

The overall goal of DEP2 is to improve efficiency and remove barriers in proteomics data analysis. DEP2 provides a reproducible tandem analysis workflow for proteomics datasets, including data processing, imputation, hypothesis testing, result visualization, and downstream biological function exploration (Fig. 1a). In DEP2, we have expanded the workflow in steps to ensure its flexibility for various quantitative results with distinct characteristics.

DEP2 accepts input in either peptide- or protein-level quantitation and supplies a reshape function to handle result files in both wide and long format tables. Following data reshaping, DEP offers three differential analysis pipelines for various proteomics results (Fig. 1a). The first pipeline, the classical approach modified from DEP, is designed for protein group quantitative results. The abundance matrix is extracted and filtered according to identification information and missing occupancy. Then, data are normalized by variance stabilizing normalization, a proven normalization method for proteomics (Valikangas *et al.* 2018), followed by data imputation and a moderated *t*-test from limma. The second pipeline focuses on PTM-specific proteomics based on modified peptide abundance, utilizing the modification sites as identifiers. The third pipeline aggregates protein-level abundance from peptide quantitative results. DEP2 integrates three protein aggregation strategies from package QFeatures (https://github.com/RforMassSpectrometry/QFeatures): Tuckey’s median polish, which calculates an overall median and sample effect (Tukey 1977); robustSummary (the summarization method in MSqRobSum), which aggregates protein intensities using robust regression (Sticker *et al.* 2020); and total sum, which simply sums up peptide quantitative data. In addition, we have constructed a workflow for RNA counts data based on DESeq2 for multi-omics data analysis (Love *et al.* 2014).

To highlight the biological information from omics analysis results, DEP2 integrates three downstream biological exploration analyses (Fig. 1a): functional enrichment, protein–protein interaction (PPI) network prediction, and expression pattern clustering. Functional enrichment involves over-representation and gene set enrichment analyses utilizing clusterProfiler (Wu *et al.* 2021) in conjunction with genome annotation databases such as gene ontology, Reactome, and MSigDB (Liberzon *et al.* 2015, Fabregat *et al.* 2018, Gene Ontology 2019). The PPI functionality constructs the network among a given protein/gene list based on STRING database (Szklarczyk *et al.* 2019). Expression pattern clustering utilizes c-means fuzzy clustering to classify regulated features in time-course or multiple-groups omics experiments.

Furthermore, we have updated the built-in Shiny application in parallel with the functionality upgrades, making it easy-to-use for researchers without programming experience. To implement the extended analysis workflow, we have restructured the app into modules and packaged different parts of the workflow as individual analysis modules (Fig. 1b). The analysis application is extendable by increasing modules to tab panels, and each component can crosstalk through global reactive values. Additionally, a log file that comprises inputs, parameters, and results can be exported after the completion of the pipeline in an omics module. In short, the application is able to execute most analysis and visualization functions in DEP2, including multi-omics comparisons, in an interactive and codeless way.

Finally, we have developed instructional materials, in the form of embedded vignettes within DEP2, to offer essential guidance for users. These vignettes demonstrate the omics analysis pipelines and post-analysis functions of DEP2 using a published research dataset of silicosis mouse model (Wang *et al.* 2022). Furthermore, we utilized a benchmark dataset created by spiking *Escherichia coli* and yeast proteomes into a human background. This benchmark dataset is employed to illustrate data import procedures for various quantitative results and facilitate a comparative analysis between DEP2 and Perseus. The vignettes, along with help documents, are also accessible on the package website.

## 3 Conclusion and discussion

Here, we launch DEP2, a package provides a comprehensive proteomics analysis toolkit, upgraded from its predecessor, DEP. DEP2 can handle a broader range of proteomics results, and seamlessly integrates biological functional analysis with differential analysis. Additionally, DEP2 incorporates a more versatile Shiny application covering functionalities in the package.

At the expense of versatility and compatibility, however, DEP2 contains a maze of options in each step. In fact, the selection of each step should consider many factors, such as experiment design, data characteristic and equipment state. Tools like StatsPro (Yang *et al.* 2022) provide an evaluation platform for statistical approaches, which may help researchers to tune analysis.
